# Adsorption and mechanism of cellulase enzymes onto lignin isolated from corn stover pretreated with liquid hot water

**DOI:** 10.1186/s13068-016-0531-0

**Published:** 2016-06-03

**Authors:** Xianqin Lu, Xiaoju Zheng, Xuezhi Li, Jian Zhao

**Affiliations:** State Key Laboratory of Microbial Technology, Shandong University, Jinan, 250100 Shandong China

**Keywords:** Lignin, Cellulase, Adsorption, Liquid hot water pretreatment

## Abstract

**Background:**

In the bioconversion of lignocellulosic substrates, the adsorption behavior of cellulase onto lignin has a negative effect on enzymatic hydrolysis of cellulose, decreasing glucose production during enzymatic hydrolysis, thus decreasing the yield of fermentation and the production of useful products. Understanding the interaction between lignin and cellulase is necessary to optimize the components of cellulase mixture, genetically engineer high-efficiency cellulase, and reduce cost of bioconversion. Most lignin is not removed during liquid hot water (LHW) pretreatment, and the characteristics of lignin in solid substrate are also changed. To understand the interactions between cellulase and lignin, this study investigated the change in the characteristics of lignin obtained from corn stover, as well as the behavior of cellulase adsorption onto lignin, under various severities of LHW pretreatment.

**Results:**

LHW pretreatment removed most hemicellulose and some lignin in corn stover, as well as improved enzymatic digestibility of corn stover. After LHW pretreatment, the molecular weight of lignin obviously increased, whereas its polydispersity decreased and became more negative. The hydrophobicity and functional groups in lignin also changed. Adsorption of cellulase from *Penicillium oxalicum* onto lignin isolated from corn stover was enhanced after LHW pretreatment, and increased under increasing pretreatment severity. Different adsorption behaviors were observed in different lignin samples and components of cellulase mixtures, even in different cellobiohydrolases (CBHs), endo-beta-1, 4-glucanases (EGs). The greatest reduction in enzyme activity caused by lignin was observed in CBH, followed by that in xylanase and then in EG and β-Glucosidase (BGL). The adsorption behavior exerted different effects on subsequent enzymatic hydrolysis of various biomass substrates. Hydrophobic and electrostatic interactions may be important factors affecting different adsorption behaviors between lignin and cellulase.

**Conclusions:**

LHW pretreatment changed the characteristics of the remaining lignin in corn stover, thus affected the adsorption behavior of lignin toward cellulase. For different protein components in cellulase solution from *P. oxalicum*, electrostatic action was a main factor influencing the adsorption of EG and xylanase onto lignin in corn stover, while hydrophobicity affected the adsorption of CBH and BGL onto lignin.

## Background

The use of lignocellulosic biomass to produce biofuels is an effective way to defuse the energy crisis [1–6]. Enzymatic hydrolysis of carbohydrate in biomass into fermentable monosaccharide is an important process in the bioconversion of lignocellulose into bioethanol. However, lignocellulose has a natural barrier against degradation (biomass recalcitrance) because of its rigid and compact structure [2], leading to inefficient enzymatic hydrolysis and low yield of fermentable sugars.

Pretreatment is the most critical step to break the biomass recalcitrance of lignocellulose prior to enzymatic hydrolysis. Many pretreatment methods have been presented and evaluated [3–7]. Liquid hot water (LHW) pretreatment has been widely investigated because of its advantages, such as environmental friendliness, low cost, no chemical addition, and potential application of dissolved hemicelluloses [8]. During LHW pretreatment, organic acid formed from dissolved hemicelluloses promotes the cleavage of the carbohydrate that reduces the degree of cellulose polymerization, especially at high pretreatment temperatures (>170 °C) [9]. Our previous studies also showed that LHW pretreatment effectively improved the enzymatic hydrolysis of some straw materials, such as corn stover and reed [3, 10], and cellulose in water insoluble solid can be highly efficiently hydrolyzed into glucose using cellulase after pretreatment. However, only partial lignin in biomass was dissolved out and the structure of the lignin in the pretreated solid residuals was also changed during LHW pretreatment [11].

Lignin is a mesh-type polymer composed of phenylpropane units produced through oxidative coupling of 4-hydroxyphenylpropanoid compounds [12]. Lignin is a “binding substance” in cell wall combining cellulose and hemicellulose, which imparts rigidity to lignocellulose and renders moisture holding capacity. Studies have shown that the presence of lignin significantly influences the efficiency of enzymatic hydrolysis of lignocellulose [13–16]. Lignin not only acts as a physical barrier limiting the access of cellulase to cellulose but also attracts cellulase, leading to nonproductive binding [14, 16]. Binding characteristic of cellulase onto lignin was affected by the chemical and physical structures of lignin. Ko et al. [11] reported that LHW pretreatment altered the lignin structure and affected the adsorption behavior of lignin toward enzymes. Pretreatment can also partly reduce physical obstruction, whereas it promotes cellulose adsorption to lignin [17, 18]. Regular variation was carried out after pretreatment with different severities, giving a good vision to analyze the adsorption behavior of lignin [16].

The adsorption behavior of cellulase onto lignin has been widely studied for mixed enzymes and purified cellulase [13–17]. Using lignin preparations isolated from organosolv-pretreated softwood, as well as seven cellulase preparations, three xylanase preparations, and one β-Glucosidase preparation, Berlin et al. found that various cellulases differed in their inhibition by lignin by up to 3.5-fold, whereas xylanases showed less variability and β-Glucosidase (Novozym 188) was least affected by lignin [14]. Ko et al. found that β-Glucosidase in CellicCtec 2 (Novozyme) demonstrated the highest adsorption to lignin isolated from LHW-pretreated hardwood [11]. Adsorption of two purified cellulases from *Trichoderma reesei*, namely, CBH I (Cel7A) and EG II (Cel5A), and their catalytic domains onto steam-pretreated softwood (SPS) and lignin were compared [19]. CBH I and its catalytic domain both exhibited higher affinity to SPS compared with EG II or its catalytic domain, and the catalytic domains of these enzymes from *T. reesei* differed essentially in their adsorption to isolated lignin and adding EG II affected the binding of CBH I obviously. The cellulase system is complex, and many different enzymes presented in cellulase systems. A suitable enzyme system is very important for high efficient degradation of cellulose. Analyzing cellulase mixture adsorption behavior, via the changes in amount of protein and enzymatic activities, hardly response adsorption behavior of specific cellulase, as well as analyze of single purified cellulase hardly represent the cellulase actual behavior in mixture. Understanding the differences of lignin adsorption on different enzyme components in cellulase mixture, and interaction between lignin and cellulase is necessary for decreasing/eliminating the negative effect of lignin on enzymatic hydrolysis of cellulose. However few comprehensive studies on adsorption of cellulase, especially of different enzyme components in the whole cellulase system, have been published.

*Penicillium oxalicum* JU-A10-T is a strain that produces cellulase with high activity; and its cellulase exhibits good potential in the enzymatic hydrolysis of lignocellulosic substrate into glucose and in cellulosic ethanol production [13, 20, 21]. Our laboratory previous study identified and classified more than 100 proteins in the cellulase enzyme of *P. oxalicum* JU-A10-T [21]. The present study attempted to determine the adsorption behaviors of different components of the integrated cellulase of *P. oxalicum* JU-A10-T onto lignin, and investigate the effect of lignin on enzyme activities and enzymatic hydrolysis of different cellulosic substrates. The study also aimed to investigate the differences in the characteristics of lignin samples isolated from corn stover pretreated under different severities of LHW. The possible cause of the change in the adsorption capacity of lignin was also interpreted from the perspective of lignin properties.

## Results

### Changes in the chemical compositions of corn stover pretreated under different severities of LHW

Table 1 shows that over 97 % hemicellulose in corn stover was hydrolyzed and removed after LHW pretreatment, and the highest amount of hemicellulose (98.62 %) was removed at a pretreatment severity of 3.9 compared with untreated corn stover. Nearly half of lignin in corn stover was dissolved out when the yield of insoluble solid substrate was considered during pretreatment. Moreover, the cellulose content in pretreated corn stover increased because of the removal of lignin and hemicellulose components. The results of enzymatic hydrolysis showed that LHW pretreatment obviously increased the conversion of cellulose into glucose during enzymatic hydrolysis. For example, the conversion of cellulose to glucose in corn stover pretreated at a severity of 3.9 increased from 30 to 68 % compared with that in untreated corn stover.

**Table 1:** Chemical compositions of corn stover untreated and pretreated with LHW of different severities (%)

Pretreatment severity	Cellulose	Lignin			Removal of lignin^a^	Hemicellulose	Removal of hemicellulose^a^
		Soluble acid	Insoluble acid	Total			
Control	33.28 ± 0.71	1.48 ± 0.04	15.09 ± 0.18	16.57	–	30.15 ± 0.71	–
S = 3.6	60.21 ± 0.87	1.00 ± 0.14	13.59 ± 0.08	14.59	52.35	1.31 ± 0.00	97.66
S = 3.9	59.72 ± 0.43	0.86 ± 0.03	13.91 ± 1.25	14.77	52.54	0.78 ± 0.04	98.62
S = 4.2	58.52 ± 0.34	0.67 ± 0.03	14.95 ± 0.69	15.62	50.24	1.6 ± 0.12	97.87

Contents of all components were based on oven dry weight of the measured substrate

^a^Removal of lignin (or hemicelluloses) = (the content of lignin or hemicellulose in pretreated corn stover × solid yield (%))/the content of lignin or hemicellulose in untreated corn stover

### LHW pretreatment-induced changes in lignin characteristics

#### Molecular weight of lignin

Table 2 shows the weight average molecular weight (Mw), number average molecular weight (Mn), and polydispersity (Mw/Mn) of lignin obtained from untreated samples and samples pretreated at different severities. Mw and Mn of lignin from pretreated corn stover were obviously higher than those from untreated corn stover, demonstrating that lignin was polymerized during LHW pretreatment. The molecular weight of lignin pretreated at a severity of 4.2 was lower than that of lignin pretreated at a severity of 3.9, indicating that lignin may be further degraded by the organic acid formed from hemicellulose hydrolysis during LHW pretreatment. In addition, the polydispersity of lignin from pretreated corn stover decreased to nearly 1, indicating that the lignin fraction in pretreated corn stover became more even.

**Table 2:** Molecular weights and polydispersity index of the lignin samples

Pretreatment severity	Mn (Da)	Mw (Da)	Polydispersity (Mw/Mn)
Untreated	19,070	22,680	1.189
S = 3.6	63,120	63,560	1.007
S = 3.9	102,800	103,200	1.004
S = 4.2	82,100	82,440	1.004

#### Surface charge and hydrophobicity of lignin

Zeta potential was used to represent the surface charge of lignin [22]. Table 3 shows that all of the lignin samples from pretreated corn stover became more negative compared with those from untreated corn stover, because some active groups carrying a negative charge (for example hydroxyl and carboxyl) in the lignin structure increased as acetic and other organic acids were released from hemicellulose hydrolysis during LHW pretreatment. Table 3 also shows that the lignin sample from corn stover pretreated at a severity of 3.9 carried the highest amount of surface charge but demonstrated the lowest lignin hydrophobicity.

**Table 3:** Zeta potential and hydrophobicity of the lignin samples

Pretreatment severity	Untreated	S = 3.6	S = 3.9	S = 4.2
potential (mv)	−13.79 ± 2.75	−18.49 ± 1.36	−31.70 ± 2.70	−21.47 ± 1.01
Hydrophobicity (mL/g)	–	0.1914	0.1114	0.1752

#### FTIR analysis of lignin

Table 4 shows the signal assignment and relative intensities in the FTIR spectra of the lignin samples. The signal intensities at 3446, 1370, and 1335 cm^−1^, which corresponded to O–H stretching vibration in the OH groups (e.g., aromatic and aliphatic—OH groups), were increased with increasing pretreatment severity. Compared with the control (untreated corn stover), however, the intensity of the signals at 1335 and 1370 cm^−1^ in lignin samples from pretreated corn stover decreased, except the signal at 1370 cm^−1^ in the lignin sample pretreated at a severity of 4.2. The intensity of the signal at 1370 cm^−1^ was prominently enhanced after LHW pretreatment at a severity of 4.2, indicating the increase in the content of phenolic hydroxyl groups in lignin samples. The signals at 1770 and 1653 cm^−1^ corresponded to carbonyl groups, and that at 465 cm^−1^ was due to the adsorption of 2 or 2, 3, 4 overlay substitution of benzene ring; all of these signals were intensified after LHW pretreatment. The intensities enhanced with increasing pretreatment severity, indicating that the oxidization and condensation reactions occurred in lignin during LHW pretreatment. The condensation reaction may increase the formation of high-molecular-weight lignin and reduce the amount of low-molecular-weight lignin, which was consistent with the results shown in Table 2. Furthermore, the vibration at 1458 cm^−1^ in –CH_3_ was caused by asymmetric C–H deformations, and the signals at 2925 and 2849 cm^−1^ were assigned to the C–H asymmetric and symmetric vibrations in methyl and methylene groups. The intensity of these three bands decreased after LHW pretreatment, suggesting that the methyl and methylene groups were removed or transformed into other chemical groups (for example C=O or –C–) during LHW pretreatment [13, 23].

**Table 4:** Signal assignment and relative intensities in the FTIR spectra of the lignin samples

Peak (cm^−1^)	Assignment	Control	S = 3.6	S = 3.9	S = 4.2
3446	O–H stretching vibration in OH groups (R–OH, Ar–OH)	14.98	14.16	15.93	22.17
2925	C–H stretching vibrations	18.19	11.72	11.90	14.02
2849		6.73	3.83	3.67	4.95
1770	Unconjugated carbonyl groups (C=O stretch)	0.70	1.22	1.56	1.65
1653	Conjugated carbonyl groups (C=O stretch)	63.01	75.08	80.54	95.00
1458	C–H vibrations in –CH3	28.40	20.01	20.91	24.43
1370	Phenolic hydroxyl groups (Ar–OH)	5.32	5.03	5.20	10.93
1335	R–OH in the lignin	6.25	3.79	3.89	4.82
465	The 2 or 2, 3, 4 overlay substitution of benzene ring	404.80	408.19	421.17	569.49

The relative intensity was calculated as the ratio of the intensity of the band to the intensity of the band at 1795 cm^−1^

*S* stands for the pretreatment severity

### Cellulase adsorption to lignin

#### Determination of protein/lignin ratio in the reaction system

**Fig. 1:**
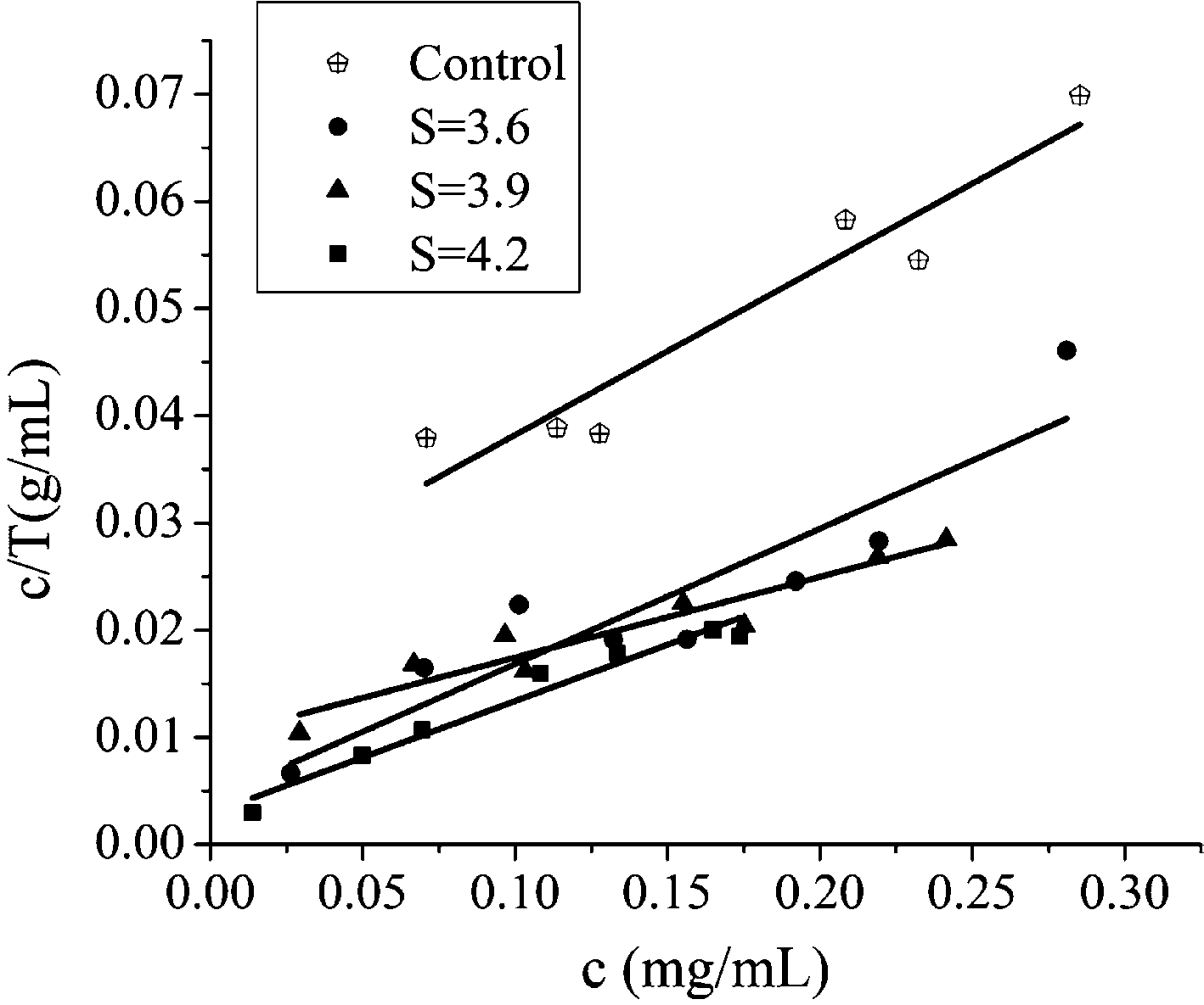
Langmuir regression line of adsorption of the lignin samples from corn stover untreated and pretreated by liquid hot water of different pretreatment severities (*S*). Fitting curve: for control: Y = 0.1564x + 0.0226 (*R* ^2^ = 0.916); for *S* = 3.6: Y = 0.0753x + 0.0099 (*R* ^2^ = 0.9074); for *S* = 3.9: Y = 0.1264x + 0.0042 (*R* ^2^ = 0.8494); for *S* = 4.2: Y = 0.1051x + 0.0029 (*R* ^2^ = 0.9646)

The dosage ratio of protein to lignin was studied to ensure sufficient protein quantity in the reaction system and system sensitivity. Using the extracellular protein in the crude cellulase solution of *P. oxalicum* JU-A10-T and the isolated enzymatic residual lignin (ERL) samples from corn stover pretreated at different severities of LHW, we estimated the maximum adsorption capacity of the lignin samples using the Langmuir adsorption isotherm (Fig. 1). The equilibrium isotherm sorption data were analyzed by the following Langmuir expression:

1 $$T = \frac{{c \cdot T_{MAX} \cdot K}}{c \cdot K + 1}$$

where *c* is the enzyme equilibrium concentration in the supernatant after adsorption, *T* is the mass of the protein adsorbed by the lignin (mg protein/g lignin), *Tmax* is the maximum adsorption mass of the lignin, and *K* is the adsorption constant.

To measure *T*_*MAX*_, we transformed formula (1) into formula (2) as follows:

2 $$\frac{c}{T} = \frac{c}{{T_{MAX} }} + \frac{1}{{T_{MAX} \cdot K}}$$

In this formula, *c* and *T* were determined through lignin adsorption experiments, and *T*_*MAX*_ was obtained by measuring the reciprocal of the fitting line slope of *c/T* and *c* (Fig. 1). The theoretical maximum adsorption values of the enzyme protein to the different lignin samples were 9.5, 8.4, 7.9, and 6.4 mg protein/g lignin at pretreatment severities of 4.2, 3.9, 3.6, and 0 (control), respectively (Fig. 1). Based on these results, 10 mg protein/g lignin was used to study the characteristics of enzyme adsorption onto lignin.

#### Effect of lignin adsorption on protein content and enzyme activities

**Fig. 2:**
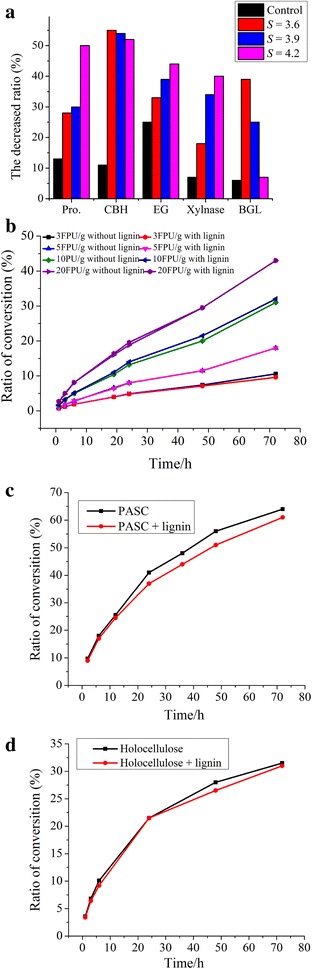
Effect of addition of different lignin samples in the system on protein content and activities of CBH, EG, BGL, and xylanase in the supernatant, and enzymatic hydrolysis of avicel, PASC, and holocelluose. **a** Effect on protein content and enzyme activities of BGL, EG, CBH and xylanase, in which reduced ratio was based on protein content and enzyme activities in the reaction system without lignin. **b**–**d** Effect on enzymatic hydrolysis of avicel, PASC, and holocellulose

After performing the adsorption experiments using different lignin samples, the protein concentration and enzyme activities in the supernatant were determined, and the reduced adsorption values were calculated based on a reaction system without lignin. As shown in Fig. 2a, compared with lignin from corn stover without pretreatment, the lignin samples from LHW-pretreated corn stover contained markedly reduced protein contents in the supernatant after adsorption with lignin. This finding indicated that LHW pretreatment obviously increased the adsorption capability of lignin toward proteins. Moreover, the adsorption capability of lignin toward protein increased with increasing pretreatment severity. The protein content in the supernatant was reduced by over 50 % after adsorption using lignin from corn stover pretreated at a severity of 4.2.

Addition of lignin into cellulase solution could also reduce the activities of enzymes, such as CBH, EG, xylanase, and BGL in the supernatant, but the effect on enzyme activities varied in different lignin samples and enzyme components. For example, CBH activity in lignin samples from LHW-pretreated corn stover sharply decreased compared with that of the control, but slight differences were observed among the three lignin samples from corn stover pretreated at different severities. Moreover, the activities of EGs and xylanase decreased with increasing pretreatment severities, whereas that of BGL demonstrated the opposite trend. Lignin adsorption exerted a relatively strong influence on CBH activity after LHW pretreatment.

Enzymatic hydrolysis was also performed using different types of cellulose substrates, avicel, phosphoric acid-swollen cellulose (PASC) and holocellulose, to further evaluate the effect of lignin adsorption on enzyme hydrolysis. Lignin from corn stover pretreated at a severity of 4.2 was used in the experiments, and the lignin dosage in the reaction system was 0.25 g lignin/g cellulosic substrate. The lignin dosage used in this study was equivalent to the lignin content in pretreated corn stover that was used in the enzymatic hydrolysis system.

As shown in Fig. 2b, regardless of cellulase dosage, the conversion of avicel into glucose slightly changed during enzymatic hydrolysis of avicel with or without lignin. Figure 2a and d showed that lignin adsorption reduced the glucose yield in the reaction system, that is, lignin adsorption reduced the enzymatic hydrolysis of PASC and holocellulose.

The holocellulose substrate from LHW-pretreated corn stover mainly contained cellulose and tiny hemicelluloses because hemicelluloses were largely removed during LHW pretreatment. EG, CBH, and BGL collectively hydrolyzed cellulose, and xylanase hydrolyzed xylan of hemicellulose in holocellulose. The amount of enzyme components in the supernatant decreased after lignin adsorption affected the enzymatic hydrolysis of holocellulose. In addition, PASC is amorphous cellulose, and its degradation is mainly affected by EG activity. Reduced EG activity by lignin adsorption weakened the enzymatic hydrolysis of PASC. Moreover, avicel is a kind of cellulose that exhibits a highly crystalline structure, and CBH is the main enzyme that hydrolyzes avicel. The slight changes in the glucose yield during enzymatic hydrolysis of avicel with and without lignin at different enzyme loading may be attributed to the adsorbed CBH remained hydrolysis activity because that binding to lignin by binding domain not the catalytic domain, but more researches need to prove that. EG nonproductive adsorption onto lignin maybe the main reason for the decreased hydrolysis of holocellulose because BGL adsorption was relatively lower.

### Adsorption profiles of different cellulase enzyme components

**Fig. 3:**
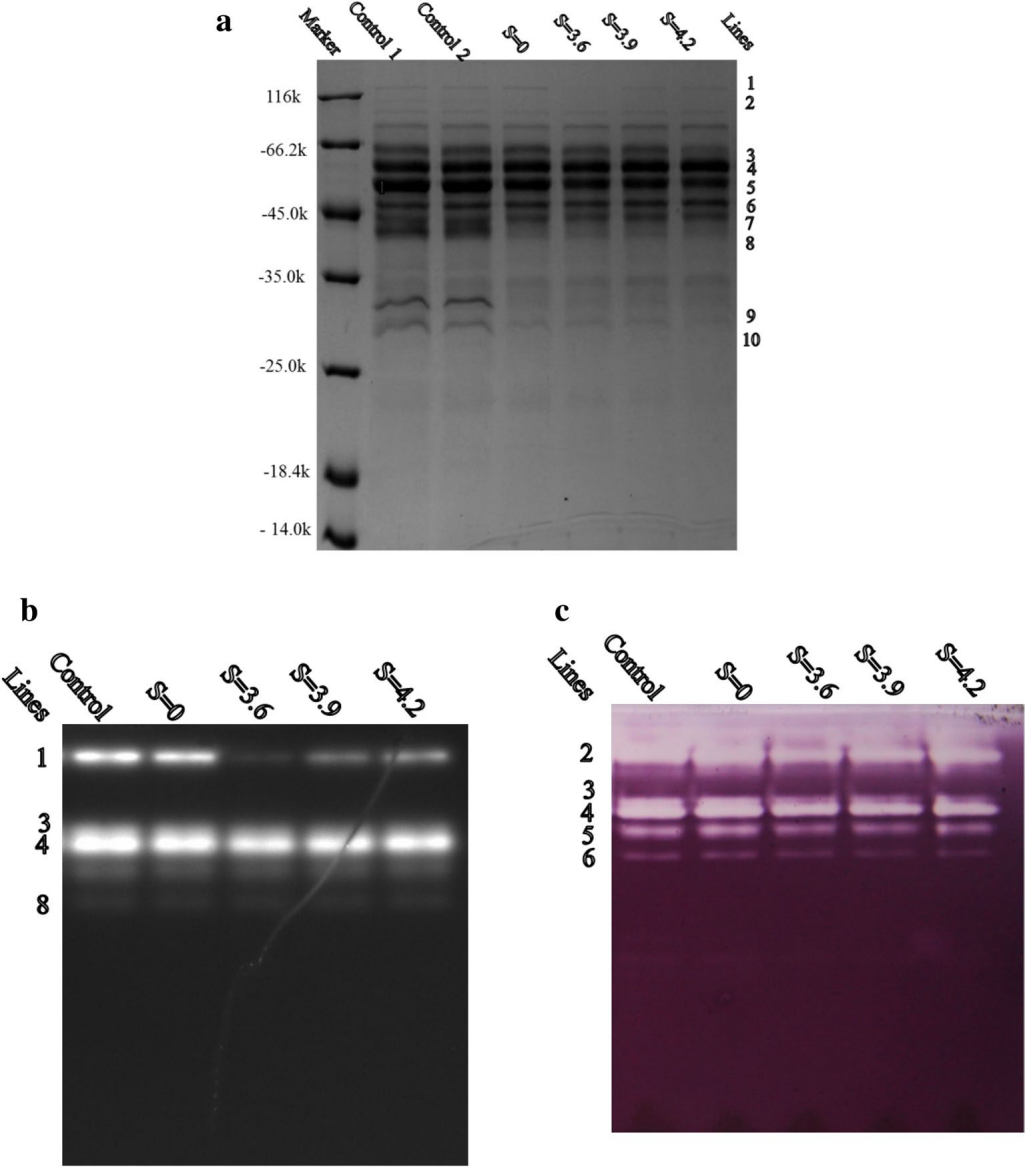
Analyze by SDS-PAGE and zymogram of proteins in the supernatant of cellulase. **a** SDS-PAGE; **b** MUC zymogram; and **c** CMC zymogram

To further investigate the adsorption profiles of specific enzyme components in the cellulase mixture, we performed Sodium dodecyl sulfate polyacrylamide gel electrophoresis (SDS-PAGE) and zymograms of protein in the liquid supernatant (Fig. 3). The protein bands, that were noticeably changed, were cut off and further analyzed by mass spectrometry, and the results are shown in Table 5. The densities of different bands in zymograms were analyzed, and their relative gray values based on control 1 were calculated (Table 6), in which control 1 was the enzyme in buffer without lignin and its gray value was set as 1 [24, 25].

**Table 5:** Proteins identified by MS in different bands in SDS-PAGE and different enzyme activities measured by MUC and CMC zymograms

Lines	Protein identified by MS	PSMs^a^	Possible main proteins in band	Enzyme activity detected by CMC	Enzyme activity detected by MUC
1	−	–	BGL1	−	+
2	Cel5C	15	Cel5C, Cel5B	+	−
	Cel5B	11		+	−
3	Cel7A-2	446	Cel7A-2	−	+
	Cel7B	69		+	−
	cel7A-1	45		−	+
	SWO	40		−	−
	Cel5C	30		+	−
4	Cel7A-2	348	Cel7A-2, Cel5B	−	+
	Cel5B	111		+	−
5	Cel6A	402	Cel6A, Cel5B	−	−
	Cel5B	157		+	−
	Cel7A-1	79		−	+
	Cel7A-2	60		−	+
6	Xylanase10A	897	Xylanase10A	+	−
7	Cel6A	1179	Cel6A	−	−
	Xylanase10A	96		−	+
8	Cel7A-2	423	Cel7A-2	−	+
	Chi18A	101		−	−
	Cel6A	91		−	−
	Xylanase10A	78		+	−
	Cel5B	56		+	−
9	Xylanase10B	1257	Xylanase10B	+	−
	Cel7A-2	190		−	+
	Abf62A	166		−	−
10	Cel61A	364	Cel61A, Cel6A	+	−
	Cel6A	283		−	−
	Fae1A	136		−	−

“+” special activity detected in the enzyme, “−” no activity detected

^a^PSMs indicate the number of peptide fragments detected for the protein analyzed by mass spectrum

**Table 6:** Relative gray value of different enzymes calculated using MUC and CMC zymograms (%)

Lines	Enzyme		S = 0	S = 3.6	S = 3.9	S = 4.2
2	EG	Cel5C, Cel5B	3.73	23.97	19.05	27.87
3		Cel5C, Cel7B	13.45	51.62	57.73	71.02
4		Cel5B	2.97	16.02	15.13	28.12
5		Cel5B	47.92	66.41	73.69	80.04
Sum			33.61	51.6	53.12	61.41
6	Xylanase	Xylanase10A	14.49	25.93	33.86	30.76
1	BGL	Bgl1	38.75	98.42	87.91	84.55
4	CBH	Cel7a-2	31.05	60.80	50.22	44.20

The enzyme without adsorption by lignin was set as control, and the gray value is the percentage relative to the gray value of the homologous line in the control

BGL1 was likely presented in line 1 of SDS-PAGE (Fig. 3a) (Mw > 116) because the molecular weight of this protein is approximately 128 [21]. It was also the only detected protein with a molecular weight higher than 116 in SDS-PAGE. This finding was further supported by the 4-methylumbeliferyl- β-d-cellobioside (MUC) (sigma) zymogram (Fig. 3b), in which a noticeably bright zone was observed at band 1. The changes in densities of the BGL1 protein band on SDS-PAGE suggested that the adsorption ability of BGL1 onto the lignin samples from pretreated corn stover obviously increased compared with that of the lignin samples from untreated corn stover, and the adsorption ability decreased with increasing pretreatment severity (the gray values were 98.42, 87.91, and 84.55 % at severities of 3.6, 3.9, and 4.2, respectively; Fig. 3b).

Cel7A-2 was the main CBH protein detected in the protein bands of lines 3, 4, and 8, and it was also found in the MUC zymogram. SDS-PAGE suggested that the Cel7A-2 protein in the supernatant was reduced after lignin adsorption, as shown in lines 3 and 8. Cel7A-2 in line 4 was obviously detected by the MUC zymogram, which also revealed that Cel7A-2 activity in the supernatant obviously deceased after lignin adsorption. By comparing lignin pretreated at a severity of 3.6 with lignin from untreated corn stover, we found that the changes in the gray value (Table 6) demonstrated that the adsorption ratio of the protein Cel7A-2 obviously increased from 31.05 to 60.8 %. However, the adsorption ratio decreased from 60.8 to 44.2 % when the pretreatment severity increased from 3.6 to 4.2. This finding was consistent with the changes in CBH enzyme activity (Fig. 3b).

Cel5B, Cel5C, and Cel7B were the main EGs detected in JU-A10-T liquor fermentation [20, 21]. The reduced ratio of total carboxymethylcellulose (CMC) enzyme activity varied from 33.61 to 61.41 % with increasing pretreatment severity from 0 to 4.2 (Table 6), and this trend was consistent with the activity detected by CMC, the total EGs activity (Fig. 2a). In addition, Cel5B and Cel5C were the main proteins detected in the band of line 2, and they were nearly undetected in SDS-PAGE after lignin adsorption. CMC activity at line 2 also markedly decreased, as revealed by the CMC zymogram. Cel7B and Cel5C were the main EGs in line 3, and their reduced gray values were from 13.45 % (untreated sample) to 71.02 % (pretreated at a severity of 4.2) as revealed by the CMC zymogram after lignin adsorption with increasing pretreatment severities. Cel5B was the main EG detected in lines 4 and 5, and its generally decreasing activity was increased by lignin adsorption under increasing pretreatment severity. The adsorption ratio of Cel5B was lower in line 4 than that in line 5. This result suggested that the protein with low molecular mass promoted the adsorption of enzyme to lignin, whereas the high-molecular-weight proteins exhibiting glycosylation inhibited lignin adsorption, as described by Kathryn et al. [26]. The CMC zymogram also suggested that Cel5C and Cel5B in lines 2 and 4 exhibited a low adsorption ratio when lignin from corn stover was pretreated at a severity of 3.9; this result was possibly attributed to the lignin characteristics.

Xylanase10A was the main enzyme in the band of line 6, and it was also detected by the CMC zymogram [27]. The reduced gray value of xylanase in line 6 varied from 14.49 to 30.76 % according to the CMC zymogram of adsorption by lignin from corn stover pretreated at different severities; lignin pretreated at a severity of 3.9 demonstrated the highest adsorption ratio of xylanase10A [24, 25].

## Discussion

Similar to the reported results on LHW pretreatment, our results showed that LHW pretreatment removed a great amount of hemicelluloses and partial lignin components in corn stover. The mechanism of LHW pretreatment has also been reported in several studies [8, 16]. However, residual lignin still exists in pretreated corn stover, and it affects subsequent enzymatic hydrolysis of cellulose by adsorbing enzymes [11]. Lignin characteristics influence the adsorption of enzymes to lignin [11, 13]. Ko. et al. reported that lignin structure was altered during LHW pretreatment and lignin isolated from more severely pretreated hardwood showed more pronounced inhibition of hydrolysis [28]. Kumar et al. also reported that in addition to lignin removal, lignin modification also played an important role in cellulase hydrolysis at low enzyme loading [18]. In the present study, lignin isolated from LHW-pretreated corn stover showed higher Mw and more uniform fragment size (lower polydispersity) compared with lignin from untreated corn stover. The changes in lignin properties promote nonproductive adsorption of enzyme proteins onto lignin [13, 14], led to protein contents in the supernatant after lignin adsorption decrease (Fig. 2a).

Hydrophobic [29, 30] and electrostatic [31] interactions were speculated to occur between cellulases and lignin. Most cellulase proteins carry a positive charge at a reaction system with pH 4.8 used in the study because the isoelectric points of proteins are higher than 4.8 [21]. Compared with lignin from untreated corn stover, lignin from pretreated corn stover displayed a higher surface charge (more negative charge, Table 3), thereby increasing the nonproductive binding of cellulase to lignin because of electrostatic attraction, which may affect the rate of lignocellulose hydrolysis. After adsorption of lignin from corn stover pretreated at a severity of 4.2, NaCl was added into the reaction system, and changes in enzyme activities of EG, CBH, BGL, and xylanase were detected to investigate the electrostatic interaction between different enzymes and lignin [11]. We found that the addition of NaCl obviously influenced xylanase activity, but it exerted a lower effect on the enzyme activities of CBH, EG, and BGL. This result suggested that electrostatic forces played an important role in the adsorption of lignin towards xylanase and weakly affected the adsorption behavior of CBH, EG, and BGL to lignin.

Difference on adsorption behaviors of lignin towards BGL from different microorganism have been reported, for example, adsorption of lignin towards BGL of *Aspergillus niger* was relative lower compared that towards BGL of *T. reesei* [11]. The adsorption of lignin towards BGL from Cellic Ctec2 was stronger than that towards BGL from Novozyme188 [32]. Ximenes et al. also reported the BGL adsorption related to the microorganism [33]. Besides BGL source, it was proved that adsorption of BGL onto lignin was also related to the feature of lignin by comparing the adsorption behavior of BGL from *P. oxalicum* to different lignin. As shown in Table 6, among the three lignin samples from corn stover pretreated at different severities, lignin from corn stover pretreated at a severity of 3.6 adsorbed the highest amount of BGL protein (adsorption ratio of 98.42 %). Table 3 showed that the lignin at a severity of 3.6 showed the highest hydrophobicity, this result was consistent with the report of Sammond et al. that hydrophobic interactions are the main force between BGL and lignin [30]. Upon comparing lignin from corn stover pretreated with LHW at a severity of 3.9 with that pretreated at a severity of 4.2, the former demonstrated lower hydrophobicity but slightly higher adsorption ratio onto BGL enzyme (87.91 vs. 84.55 %) possibly because of its greater negative charge (Table 6). Together, hydrophobicity and electrostatic interactions affected the lignin adsorption behavior and reduced the capability of lignin to adsorb BGL enzyme under increasing pretreatment severity.

We also found that a certain amount of EG and CBH enzymes was adsorbed onto the lignin samples, and the ability of lignin from LHW-pretreated corn stover to adsorb these enzymes increased compared with that from untreated biomass. However, different change occurred on adsorption of lignin between CBH and EG during LHW pretreatment, adsorption of total CBH proteins by lignin weakened with increasing pretreatment severity, but the capability of lignin to adsorb EG protein enhanced with increasing pretreatment severity. Ko et al. observed that as the pretreatment severity increased, the ratio of adsorption of CBH had no obvious tendency while EG was slightly lowered by 9.6 % [11], this was thought be due to major cellulase from *T. reesi* have negative or neutral charges at pH 4.8 (pI 4.7 and 4.9 for EG I and EG II respectively). Compared with CBH, the cellulose binding domain (CBD) of the EG enzyme was not necessary for the adsorption of EG enzyme onto lignin, whereas catalysis domain (CD) played a significant role in the adsorption of EG to lignin [19]. EG carried a positive charge in the adsorption system (pH 4.8) used in the present study because its isoelectric point (pI) was higher than 4.8. For example, pI values of 6.4 for Cel5C and 5.8 for Cel5B were reported by Wei et al. [21]. Based on above, we speculated that electrostatic interactions play an important role in the adsorption of the EG enzyme onto lignin. Moreover, lignin from corn stover pretreated at a severity of 3.6 carried a lower negative charge compared with the two other lignin samples from pretreated corn stover, leading to the relatively weak adsorption of EG by this lignin. A slightly increased adsorption capability of lignin at a severity of 3.9 compared with that at a severity of 4.2 was attributed to the lignin’s relatively low hydrophobicity. Based on the changes in gray value of proteins revealed by the CMC zymogram, more low-molecular-weight EG protein could be adsorbed by lignin (Table 6), and the reason behind this phenomenon will be investigated in our future work.

For the CBH enzyme, the CBD was necessary for the CBH protein to bind to lignin [19], and hydrophobic binding was the main force between CBD and lignin [34]; thus, the reduced hydrophobic properties of lignin from pretreated corn stover caused by increased pretreatment severity was possibly the main reason for the reduced capability of lignin to adsorb the CBH enzyme. The adsorption of CBH by lignin at a severity of 3.9 was slightly higher than that at a severity of 4.2 because lignin possessed a more negative charge at a severity of 4.2 (Table 6), and the electrostatic action between the lignin sample with CBH protein increased.

A previous study showed that the increase in the amount of phenolic hydroxyls in lignin is related to increased enzyme binding/inhibition capacity [35]. Using lignin model compounds, Pan et al. [36] found that phenolic hydroxyl groups play an important role in lignin–enzyme interactions. Ximenes et al. [35] reported that the addition of soluble phenolic compounds can further inhibit enzyme activity. In the present work, high content of phenolic hydroxyl in lignin from biomass pretreated with LHW at a severity of 4.2 may increase the capacity of lignin to adsorb enzymes. The contents of nonconjugated carbonyl groups (1770 cm^−1^) and conjugated carbonyl groups (1653 cm^−1^) increased after LHW pretreatment, which were beneficial for cellulase adsorption to lignin by increasing the negative charge on the lignin surface. The reduced contents of methyl and methylene groups in the lignin structure may also affect the hydrophobicity of lignin, as well as the adsorption behavior of lignin toward cellulase.

## Conclusions

LHW pretreatment improved the enzymatic digestibility of corn stover, but the capability of residue lignin to adsorb cellulase was obviously enhanced after pretreatment. The changes in hydrophobicity and surface charge (electrostatic action) of lignin and in the functional groups in the lignin structure after pretreatment at different severities may play important roles in the different adsorption behaviors of various lignin samples toward different protein components of cellulase mixture. In *P. oxalicum* cellulase mixture, electrostatic interaction played an important role in EG and xylanase adsorption onto lignin and the hydrophobicity mainly affected the adsorption of lignin on CBH and BGL. The CBD of CBH played an important role in protein-lignin binding behavior because in the mixture CBH had higher adsorption capability to lignin while adsorbed CBH barely affected the hydrolysis of avicel, but further research is needed.

## Methods

### Materials

*Penicillium oxalicum* JU-A10-T was stored in the laboratory, and crude cellulase was produced through liquid fermentation of *P. oxalicum* JU-A10-T according to a previously reported method and culture conditions [19]. Corn stover was obtained from Quanlin Paper Limited Company, Shandong Province, China. Avicel was purchased from Sigma–Aldrich, St. Louis, MO, USA. Holocellulose from LHW-pretreated corn stover was prepared according to the method described by Eom et al. [37]. PASC was prepared from avicel according to the method reported previously [13, 38].

### LHW pretreatment

LHW pretreatment was performed in a 1.5 L digester according to the literature [3]. The ratio of solid to liquid in the pretreatment was 10:1. The pretreatment temperature was set at 190 °C, and pretreatment times were 10, 20, and 40 min. Pretreatment severity (S) was calculated according to formula (3):

3 $${\mathbf{logR}}_{{\mathbf{0}}} = \, {\mathbf{log}} \, \left\{ {{\mathbf{t}} \times {\mathbf{exp}} \, \left[ {\left( {{\mathbf{T}} - {\mathbf{100}}} \right)/{\mathbf{14}}.{\mathbf{75}}} \right]} \right\}$$

where LogR_0_ is the pretreatment severity, t is the pretreatment time (min), and T is the pretreatment temperature (°C) [39]. After LHW pretreatment, the residual solid and pretreatment liquor were separated through filtration. The residual solid was washed with tap water until neutral pH was achieved and then stored at 4 °C for subsequent experiments.

### Enzymatic hydrolysis

Enzymatic hydrolysis of corn stover with or without pretreatment was performed in 50 mM acetate buffer (pH 4.8) at 10 % substrate consistency and then incubated at 50 °C under 150 rpm for 72 h. The enzyme loading was 15 FPU/g dry solid substrate. After hydrolysis, the solution was separated by centrifugation, and the supernatant was used for high-performance liquid chromatography (HPLC) (Shimadzu, Kyoto, Japan) analysis.

### Preparation of lignin

Milled wood lignin (MWL) samples from untreated and pretreated corn stover were extracted using aqueous dioxane (96 %) according to a reported method [13] and used to analyze lignin characteristics, because MWL is representative to native lignin in biomass substrate. ERL was prepared according to the method described by Berlin et al. [14], briefly carbohydrates in untreated and pretreated corn stover were thoroughly hydrolyzed by repeated enzyme treatment until the content of residual sugar in lignocellulosic substrate was lower than 2 % (on dry weight of solid substrate), and then proteinase was used to remove the cellulase enzyme in the substrate. ERL is structurally similar to MWL, but it produces a relatively higher yield [16, 25], and it was used in adsorption and enzymatic hydrolysis experiments.

### Analysis of lignin properties

The functional groups in the lignin structure were analyzed using FTIR (Nexus, Thermo Nicolet, Thermo Fisher Scientific, Waltham, MA, USA) with KBr pellets at a range of 400–4000 cm^−1^. The zeta potential of lignin was measured at 25 °C in sodium acetate-acetic acid (NaAc-HAc) buffer (0.05 M, pH 4.8) using Zeta Potential Analyzer [15, 22]. Mw and Mn of lignin were determined using gel permeation chromatography with dimethylformamide.

Rose bengal solution was used to measure the hydrophobicity of lignin according to the method described by Li et al. [29]. In brief, different amounts of lignin were incubated with a certain amount of rose bengal in 0.05 M NaAc-HAc buffer (pH 4.8) at 50 °C and centrifuged at 200 rpm for 2 h; free rose bengal in the supernatant was subsequently determined at 543 nm after centrifugation. The ratio of free dye to the adsorbed dye versus lignin concentration was plotted, and the surface hydrophobicity of lignin was defined as the trend line slope.

### Adsorption of cellulase enzyme onto lignin

All adsorption tests were performed in triplicate. Lignin and cellulase were added into a 1 mL reaction system and incubated in 50 mM acetate buffer (pH 4.8) at 50 °C for 48 h under 50 rpm. After incubation, the supernatant was separated by centrifugation and used to analyze enzyme activity and protein content. The adsorbed protein was calculated by the change of  free protein in the supernatant before and after adsorption. For Langmuir adsorption, the quantified lignin and different concentrations of cellulase were used in the experiments. The cellulase dosage in the reaction system for other adsorption experiments was 10 mg protein/g lignin.

For enzymatic hydrolysis of avicel, PASC, and holocelluose, 0.25 g lignin/g substrate was added, and hydrolysis experiments were performed using a cellulase dosage of 3 FPU/g substrate at 10 % of solid consistency and 50 °C under 150 rpm for 72 h. During enzymatic hydrolysis, the solution sample was extracted at certain intervals and then centrifuged. The supernatant was used to analyze the glucose content using HPLC.

### Analysis methods

The chemical components of corn stover and pretreated corn stover were analyzed according to the methods of National Renewable Energy Laboratory (USA) [40]. Briefly, the biomass was firstly extracted by anhydrous ethanol, and then it was completely hydrolyzed to different monosaccharide such as glucose and xylose by two stage acidic hydrolysis with 72 and 4 % sulfuric acid respectively. The filtered acid-hydrolysis liquid was neutralized with powder Ba(OH)_2_ and then centrifuged for 15 min, and the glucose content in the supernatant was measured using an SBA-40C biological sensor analyzer (Biological Institute of Shandong Academy of Science, Shandong Province, China). Cellulose content was calculated using formula (4). Xylose content was determined using HPLC with a refractive index detector (Shimadzu) on an Aminex HPX-87P column (Bio-Rad, Hercules, CA, USA) running at a flow rate of 0.5 mL/min at 78 °C, with water as eluent. Hemicellulose content was calculated using formula (5).

4 $${\text{Cellulose content}}\,\left( \% \right)\, = \,\frac{{{\text{Glucose released from acid hydrolysis}}\, \left( {\text{mg}} \right)\, \times \, 0.9 }}{{{\text{sample weight}}\, ({\text{mg}})}}\, \times \,100\,\%$$

5 $${\text{Hemicellulose content}}\,\left( \% \right)\, = \,\frac{{{\text{Pentose released from acid hydrolysis }}\,\left( {\text{mg}} \right)\, \times \,0.88 }}{{{\text{sample weight }}\,({\text{mg}})}} \, \times \,100\,\% .$$

The activities of CBH, EG, BGL, and xylanase were assayed according to procedures described in the literature [13]. In brief, using 1 % pNPC (with d-gluconic acid-d-lactone as inhibitor), CMC-Na, salicin, and xylan (Sigma-Aldrich, St. Louis, MO, USA) as substrate for CBH, EG, BGL and xylanase respectively, we conducted the reaction in 50 mM NaAc-HAc buffer of pH 4.8 at 50 °C for 30 min. The amount of enzyme used to produce 1 µmol product per minute was defined as a unit of enzyme activity. The Bradford method was used to determine the protein concentrations with BSA (Sigma–Aldrich) as a standard [31].

### Protein analysis

SDS-PAGE assay was performed in a 12 % polyacrylamide gel (Bio-Rad). After staining with Coomassie Blue R-250 (Bio-Rad), the adsorption profiles of different proteins on the gels were determined by measuring the changes in band density using the densitometry function of Alpha Imager TM2200 (Alpha Innotech). The obviously changed protein bands were cut off, and the proteins were identified using mass spectrometry (LTQ Orbitrap XL, Thermo Fisher, USA) [24].

The zymograms were used to visualize the changes in the activities of the enzymes in the supernatant. CMC zymography was performed as described by Joynson et al. with slight modifications [27]. In brief, 12 % polyacrylamide SDS gel containing 0.2 % CMC was used as substrate for activity staining, and the sample was mixed with sample buffer (containing SDS). The gels were run at 89 V for 30 min and turned to 138 V for 1.5 h. The 5 % triton X-100 solution was used to remove SDS by washing the gels four times (each time for 30 min). The gel was then washed twice with acetate buffer (pH 4.8) for 10 min at 4 °C and then incubated for 30 min at 50 °C. Following incubation, the gels were stained with 0.1 % (w/v) Congo red for 1 h and then de-stained by 1 M NaCl solution overnight. To enhance the visualization of clear zones, acetic acid was added dropwise to the NaCl solution containing the gel, turning the Congo Red from red to deep purple. Detection of CBH activity was performed directly on the polyacrylamide gels using MUC (4-methylumbeliferyl-β-d-cellobioside) as substrate (Sigma) [24]. The gels washed with acetate buffer were directly immersed in the buffer containing 1 mM MUC and incubated at 50 °C for 1 h, then immediately visualized under UV illumination.

## Abbreviations

LHW: liquid hot water pretreatment
BGL: β-Glucosidase
CBH: cellobiohydrolase
EG: endoglucanase
FTIR: Fourier transform infra-red spectroscopy
GPC: gel permeation chromatography
HPLC: high performance liquid chromatography
Mn: number average molecular weights
Mw: weight average molecular weights
PASC: phosphoric acid-swollen cellulose
SDS-PAGE: sodium dodecyl sulfate polyacrylamide gel electrophoresis
*P. oxalicum*: *Penicillium oxalicum*
SPS: steam pretreated softwood
ERL: enzymatic residual lignin
MUC: 4-methylumbeliferyl-β-d-cellobioside
CMC-Na: sodium carboxymethylcellulose
CBD: cellulose binding domain
CD: catalysis domain
MWL: milled wood lignin
BSA: bull serum albumin
